# Regulation of Inflammatory Cytokine Storms by Mesenchymal Stem Cells

**DOI:** 10.3389/fimmu.2021.726909

**Published:** 2021-07-29

**Authors:** Lu Wang, Yun Li, Moyan Xu, Zihui Deng, Yan Zhao, Mengmeng Yang, Yuyan Liu, Rui Yuan, Yan Sun, Hao Zhang, Heming Wang, Zhirong Qian, Hongjun Kang

**Affiliations:** ^1^Medical School of Chinese PLA, Beijing, China; ^2^Department of Critical Care Medicine, The First Medical Center, Chinese PLA General Hospital, Beijing, China; ^3^Health Care Office, Chinese PLA General Hospital, Beijing, China; ^4^Department of Basic Medicine, Graduate School, Chinese PLA General Hospital, Beijing, China; ^5^School of Public Health, Capital Medical University, Beijing, China; ^6^Institute of Biomedical and Pharmaceutical Sciences, Guangdong University of Technology, Guangzhou, China; ^7^Scientific Research Center, The Seventh Affiliated Hospital, Sun Yat-sen University, Shenzhen, China

**Keywords:** mesenchymal stem cells, cytokine storm syndrome, COVID-19, immune system, sepsis

## Abstract

Mesenchymal stem cells (MSCs) have been widely used in preclinical and clinical trials for various diseases and have shown great potential in the treatment of sepsis and coronavirus disease (COVID-19). Inflammatory factors play vital roles in the pathogenesis of diseases. The interaction between inflammatory factors is extremely complex. Once the dynamics of inflammatory factors are unbalanced, inflammatory responses and cytokine storm syndrome develop, leading to disease exacerbation and even death. Stem cells have become ideal candidates for the treatment of such diseases due to their immunosuppressive and anti-inflammatory properties. However, the mechanisms by which stem cells affect inflammation and immune regulation are still unclear. This article discusses the therapeutic mechanism and potential value of MSCs in the treatment of sepsis and the novel COVID-19, outlines how MSCs mediate innate and acquired immunity at both the cellular and molecular levels, and described the anti-inflammatory mechanisms and related molecular pathways. Finally, we review the safety and efficacy of stem cell therapy in these two diseases at the preclinical and clinical levels.

## Introduction

Sepsis is a clinical syndrome characterized by a severe systemic inflammatory response induced by infection, trauma and secondary injury to tissues and organs. The main symptoms are a sharp drop in blood pressure, fever, diarrhea, and disseminated coagulation. Septic shock is the most common cause of death in patients in intensive care (1). COVID-19 is an acute respiratory infection caused by novel coronavirus (SARS-CoV-2) infection (2, 3). Most patients with severe COVID-19 have acute respiratory distress syndrome (ARDS). With acute myocardial injury, shock, arrhythmia, and acute renal injury, complications can develop, and patients eventually die of multiple organ dysfunction (4–6). COVID-19 and ARDS are caused by microbes invading the body rapidly, replicating and spreading, inducing a strong immune response, and excessive activation of the immune system in response to the infection results in the production of many inflammatory factors, in which is known as Cytokine storm syndrome (CSS) (7, 8) Therefore, understanding the underlying mechanism of inflammatory cytokine storms is key to developing new treatments for diseases.

Cytokines and chemokines play important roles in the immune response and immunopathological damage associated with virus-mediated diseases. When the virus invades the body and enters the bronchi and alveoli through the upper respiratory tract, the body stimulates an immune response. Macrophages produce cytokines and inflammatory chemokines at the infection site and induce the activation of lymphocytes and neutrophils, which phagocytose and isolate the virus (9). This process is necessary in the early stage of inflammation caused by viral infections, which has a positive effect on controlling viral infections. However, a maladjusted and excessive immune response will cause immune intensification, which will lead to the overexpression of inflammatory factors in patients, resulting in a cytokine storm (10–12). The essence of a cytokine storm is an excessive immune response caused by various stimuli, which was first used to describe graft versus host disease (13). During a cytokine storm, the roles of cytokines in the immune response are complex, showing characteristics of a network, and these cytokines play roles in inducing local inflammation, promoting disease progression, regulating cellular and molecular immune responses, eliminating infection, and regulating tissue repair (14). Cytokine storms can cause serious damage to the body, such as hyaline membrane formation, diffuse alveolar injury, and fibrin exudation, and then accelerate lung injury, while serious lung injury and cytokine storms in the circulatory system further induce multiple organ dysfunction and injury throughout the body (15, 16). At present, it is believed that the cause of severe pneumonia is not the virus itself but the excessive immune response induced by infection, and an unbalanced cytokine response in the body is an important cause of pneumonia and acute lung injury (17). Cytokine storms are also important factors leading to multiple organ failure and poor prognosis in other diseases caused by pathogenic coronavirus infection (18).

MSCs are pluripotent stem cells originating from the early mesoderm and ectoderm that have the advantages of abundant availability, multidirectional differentiation potential, high amplification efficiency *in vitro* and low immunogenicity (19). In addition, MSCs have powerful immunomodulatory and anti-inflammatory functions, which can regulate the innate immune and acquired immune systems (20); MSCs also exert bidirectional regulation of the immune system (20, 21) and can not only inhibit the overactivated immune system (21, 22) but also repair defective immune function (23). In summary, MSCs can alleviate and repair the lung injury caused by an excessive immune response to viruses and reduce the risk of CSS and ARDS by regulating the function of immune cells, reducing the levels of inflammatory secretion, increasing the levels of anti-inflammatory factors and the secretion a variety of cellular growth factors. Therefore, MSCs are considered to be an effective way to treat sepsis and COVID-19 (24).

## Anti-Inflammatory Effects and Mechanism of MSCs

### Mechanism of Inflammatory Storms

In the early stage of viral infection, the stimulation of the lungs leads to the recruitment and activation of a variety of inflammatory cells, and a large number of cytokines and inflammatory chemokines are released. In the early stage of stimulation, “early reactive cytokines” such as tumor necrosis factor-α (TNF-α) and interleukin-1β (IL-1β) are secreted rapidly and reach a peak within a few hours; then, anti-inflammatory cytokines are secreted to regulate the degree of the inflammatory response so that the body can not only remove harmful stimuli but also maintain cellular homeostasis (25–27). However, when the inflammatory balance is destroyed, early reactive cytokines can further trigger the activation and release of a series of cytokines, such as interleukin-2 (IL-2), interleukin-6 (IL-6), interleukin-8 (IL-8), interleukin-12 (IL-12), macrophage inflammatory protein-1α (MIP-1α), MIP-1β and others, resulting in an uncontrolled inflammatory response (28, 29). The mechanism of the excessive immune stimulation caused by coronavirus infection is still controversial, but through the study animal models and samples from patients with SARS and Middle East respiratory syndrome (MERS), which are both caused by coronavirus infection, some key factors that may lead to cytokine storms have been found (30).

### Mechanism of the Therapeutic Effects of MSCs in the Inflammatory Environment

MSCs have the advantage of low immunogenicity and can exert immunomodulatory effects through direct contact with immune cells and the secretion of soluble factors. MSCs also tend to be targeted to inflammatory sites to play anti-inflammatory roles (31). At the site of tissue injury, inflammatory mediators produced by the local microenvironment during acute inflammation activate the differentiation of monocytes into M1 macrophages. M1 macrophages produce a large number of proinflammatory factors, including interferon alpha (IFN-α) and TNF-α, and mainly engulf and digest dead cells and pathogenic microorganisms that invade the body (32). These proinflammatory agents activate resting MSCs and induce immunosuppressive phenotypes. After being activated, MSCs respond to inflammatory stimulation and produce chemokines to recruit lymphocytes to the damaged site. Anti-inflammatory MSCs produce a large number of immunosuppressive molecules, including indoleamine 2,3-dioxygenase (IDO) and nitric oxide (NO), to inhibit the killing effect of lymphocytes recruited to the inflammatory site (33), thus reducing the overall level of the immune response. MSCs repair tissue damage by producing growth factors and other supportive factors.

MSCs can regulate adaptive immune cells. Adaptive immunity refers to the immune response mediated by T and B lymphocytes stimulated by foreign substances. In contrast to innate immunity, MSCs (34)can inhibit the proliferation and activation of T lymphocytes (including helper T lymphocyte 1 (Th1 cells), Th17 cells and cytotoxic T lymphocytes) (35–37). T lymphocytes are arrested in the G0/G1 phase by inhibiting cell division, which can occur in two main ways: 1) through the interaction between cells; and 2) through soluble cytokines,(transforming growth factor-β(TGF-β), hepatocyte growth factor (HGF), IL-6, IL-10, prostaglandin E2(PGE_2_), NO, IDO, etc.) secreted by MSCs that act on T lymphocytes to inhibit their proliferation and activity (38, 39). MSCs regulate B lymphocytes by inhibiting the proliferation and differentiation of B lymphocytes, preventing B lymphocyte differentiation into plasma cells, reducing the expression of chemokine receptor 4 (CXCR4),chemokine receptor 5 (CXCR5), chemokine receptor 7(CXCR7) and their corresponding ligands chemokine receptor 12(CXCL12)and chemokine receptor 13(CXCL13), and reducing the production of Immunoglobulin M(IgM),Immunoglobulin G(IgG) and Immunoglobulin A(IgA) (40, 41). The inhibitory effect of IFN-γ on B lymphocytes may occur through the induction of IDO expression in MSCs, which can inhibit the proliferation of B lymphocytes through tryptophan (42).

MSCs regulation of the inflammatory immune response mainly includes the regulation of innate immunity and the acquired immune response. Inherent immunity gradually developed during the evolution of organisms, and it is the first line of defense against pathogens. A large number of studies have confirmed that MSCs can participate in intercellular immunoregulation through paracrine mechanisms, and these mechanisms have been verified in disease models such as acute respiratory distress syndrome, pneumonia and sepsis. Paracellular secretion refers to a process by which cells secrete regulatory factors through extracellular vesicles (EVs) (43, 44), which diffuse to intercellular spaces or tissue fluids and regulate adjacent target cells. EVs are important intercellular transporters that play important roles in the transmission of substances and signal communication between cells. The extracellular vesicles produced (45) by MSCs have immunoregulatory effects. MSCs mediate the balance of the immune response through paracrine signaling, which ultimately promotes the downregulation of the local inflammatory response and reduces tissue inflammatory injury. The types of innate immune cells regulated by MSCs through paracrine signaling include natural killer (NK) cells, neutrophils, macrophages and dendritic cells. Immunosuppressive factors produced by paracrine signaling can downregulate the expression of the NK cell surface-activated receptors NKG2D, NKp30 and NKp44 and the release level of granzyme b (46–49). Neutrophils play an important role in acute inflammatory reactions, and MSCs can still significantly inhibit neutrophil apoptosis by secreting IL-6 (50). MSCs can also recruit neutrophils to the inflammatory site by secreting IL-8 and macrophage migration inhibitory factor (MIF) and enhance the ability of neutrophils to clear pathogens (51).

### Immunoregulatory Mechanism of MSCs

MSC regulation of the inflammatory immune response mainly includes the regulation of nonspecific and specific immune responses. Both responses have their own mechanisms but are related to each other. Nonspecific immunity gradually develops *via* evolution and is one of first lines of defense against pathogen invasion. Viral invasion of airway epithelial cells starts through the cell membrane or pattern recognition receptors (PRRs), which identify viral genomic DNA, ssRNA, proteins and other components, activate nonspecific immunity, and participate in the production of cytokines such as type I interferons, IL-12, chemokines, IFN-gamma, IL-6 and TNF-α (52). During acute viral infections, type 1 interferons produced by macrophages, dendritic cells and mononuclear cells can play a role in viral replication and functional protein production and have direct effects on viral proteins to inhibit protein replication; type 1 interferons can also activate NK cells (NK cell cytotoxicity), and activated NK cells can produce IFN-gamma and enhance class helper T lymphocytes 1 (Th1) cell activity to enhance cytotoxic T lymphocytes (CTLs), NK cells and macrophages (53, 54). If the expression of IFN-stimulating genes in nonspecific immune cells is upregulated by the pathogenic virus through a cascade reaction, a chain reaction will occur, leading to the occurrence of a cytokine storm. Endothelial cells expressing sphingosine-1-phosphate receptors have been indicated to play a key coordinating role in cytokine storms (55). Moreover, TNF-α is also an important inflammatory factor that can induce lung endothelial cell activation and granulocyte and white blood cell(WBC) migration. TNF-α is considered a key proinflammatory factor in cytokine storms and may lead to new coronavirus infections after symptom exacerbation and pathological damage; therefore, inhibiting TNF-α a therapeutic strategy for treating cytokine storms (56). In addition, the secretion of chemokines can attract more nonspecific immune cells to enter infected tissues, leading to increased secretion of cytokines and exacerbation of cytokine storms and lung injury (57). Adaptive immunity refers to the immune response mediated by T and B lymphocytes, which are stimulated by foreign substances. Unlike innate immunity, has adaptive immunity exhibits specificity, tolerance and memory. MSCs regulate T lymphocytes by inhibiting the proliferation and activation of Th1, helper T lymphocytes 17 (Th17) and CTLs (58). The key to specific immune activation involves virus-mediated induction of Th1 cell secretion of cytokines to recruit mononuclear macrophages into the lung parenchyma (59). Depending on the cytokines secreted, Th cells are divided into Th1 and lymphocytes T2 (Th2) cells, and IFN-gamma is the most important Th1 cytokine. Th2 cells can secrete the cytokines IL-4, IL-5, IL-6, and IL-10 and promote the humoral immune response; during coronavirus infection, high levels of IL-10, cytokine storm occurrence are positively related to the severity of lung inflammation (60). During the development of coronavirus-induced pneumonia, specific immune factors and nonspecific immune cytokines are produced, and chemokine-mediated induction of proinflammatory and anti-inflammatory factors affects the infection site, leading to extensive lung tissue edema, inactivation of the alveolar surface, pulmonary capillary leakage, acute lung injury, and cytokine storms that further induce inflammation and damage to the structure of the lung alveolae caused by hypoxemia, eventually progressing to ARDS respiratory failure, and death (61). Thus, cytokine storms are the main mechanism of death in COVID-19 patients.

### Signaling Pathways/Molecules Related to MSCs-Mediated Regulation of Inflammation

**Figure 1:**
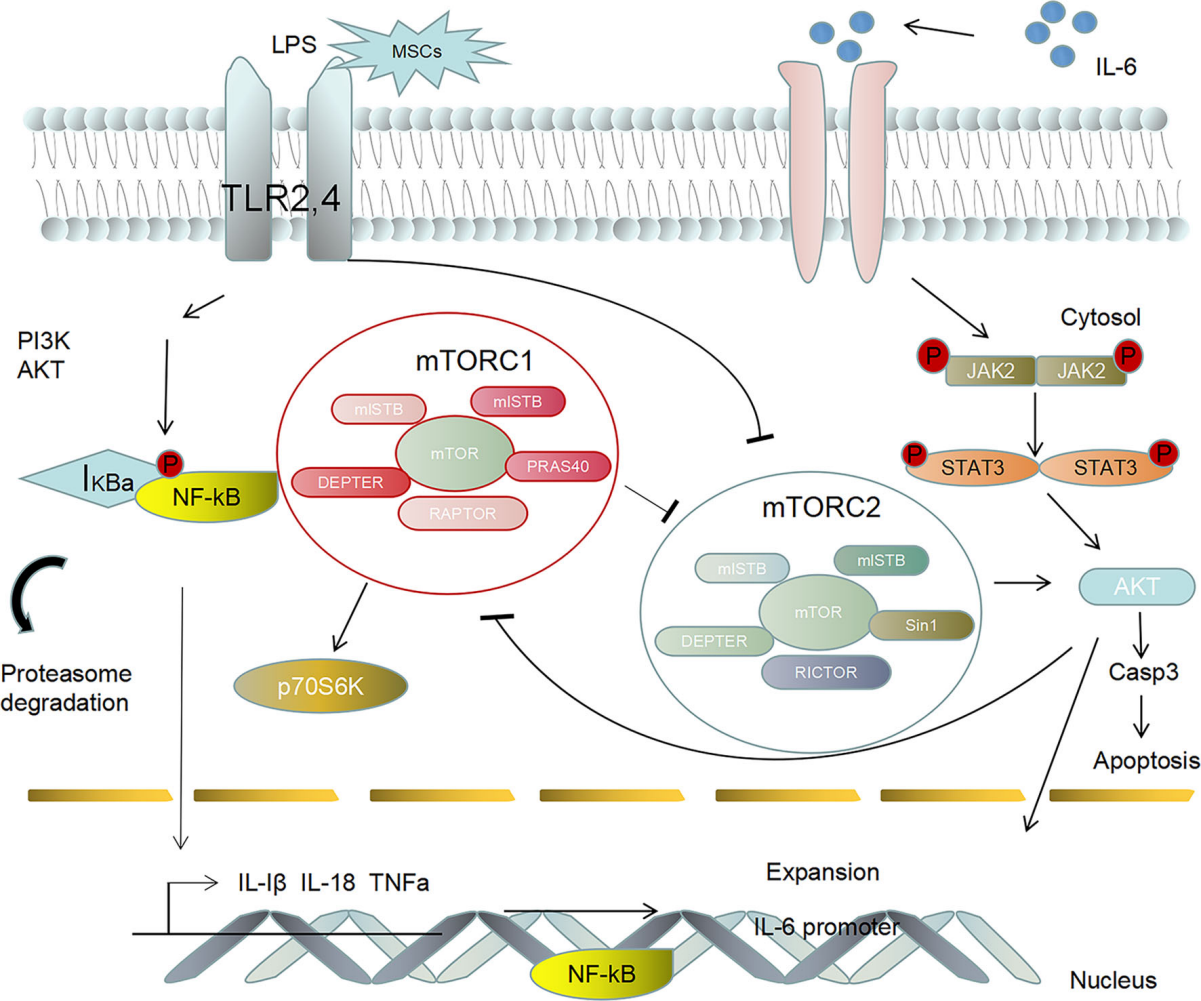
MSCs activate the adaptive immune response by recognizing Toll-like receptors (TLR2, 4), thus activating the PI3K/AKT and NF-κB signaling pathways to regulate the inflammatory response. Furthermore, MSCs can regulate the abnormal secretion of the anti-inflammatory cytokine IL-6, thus promoting the activation of the downstream signaling factors JAK2 and STAT3. The phosphorylation of signaling pathway factors induces NF-κB activation to promote the expression of IL-Iβ, IL-18, TNF-α and IL-6 in the cytoplasm to the nucleus, which leads to an inflammatory storm.

MSCs are involved in a variety of cell signaling pathways associated with inflammatory responses. Studies have shown that coculture with MSCs can reduce the activation of nuclear factor κ-B (NF-κB) in target epithelial cells under inflammatory serum conditions, and MSCs secrete sTNFR1, thereby partially improving the inflammatory response in animal models after MSCs transplantation (62). Bone marrow mesenchymal stem cells (BMSCs) can reduce alveolar macrophages (AM) apoptosis by downregulating the p-GSK-3b and β-catenin pathways in AMs, thus slowing ARDS caused by acute lung injury (ALI). BMSCs can reduce the tissue levels of NF-κB, STAT-3, TNF-α, IL-1β, iNOS, and Bax and increase the anti-inflammatory cytokine IL-10 and the anti-apoptosis biomarker B-cell lymphoma-2 (Bcl-2) to achieve anti-inflammatory and anti-inflammatory effects in the context of sepsis (63). An interesting study showed that BMSCs inhibit septicemia by inhibiting the activation of macrophage NLRP3 inflammatory bodies by enhancing mitochondrial phagocytosis and reducing mitochondrial reactive oxygen species (ROS) levels (64). Other researchers have shown (65) that mesenchymal stem cells MSCs have anti-inflammatory properties and can express genes and secrete factors that improve the survival rates of individuals with sepsis. The choline anti-inflammatory pathway (CAP) is mediated by α7 nicotinic acetylcholine receptor (α7nAChR), which plays an important role in controlling systemic inflammation. Overexpression of HO-1 in human bone marrow mesenchymal stem cells enhances the therapeutic effect of bone marrow mesenchymal stem cells on acute kidney injury, which may be related to the activation of the JAK/stat3 signaling pathway (66). The immunomodulatory effects of bone marrow mesenchymal stem cells are related to the inhibition of mTORC1-p70S6K and the activation of the mTORC2-Akt signaling pathway. The possible mechanism involved regulating the expression of inflammatory cytokines by activating mTORC2-Akt and inhibiting the mTORC1-p70S6K signaling pathway (67). **Figure 1** shows that MSCs regulate the immune response through cellular pathways and secrete related immune factors that participate in the process of inflammatory storms during treatment.

## Therapeutic Effects of MSCs After Pretreatment and Genetic Modification

### Regulatory Effects of Pretreated MSCs on Inflammation

MSCs-based treatments are currently facing some limitations. Differentiation is not stable, and there have been some instances of direct differentiation into cancer cells or uncontrolled stem cell replication. Recent studies have shown that pretreatment, genetic modification and optimization of MSC culture conditions can improve the value of MSCs *in vivo*. The key to their ability and function *in vivo (* 68) is that pretreated MSCs have significantly improved survival rates, increased differentiation effects, improved paracrine functions, and improved abilities to repair damaged tissues. Studies have shown that MG132, which is secreted by human embryonic mesenchymal stem cells (hESC-MSCs) pretreated with trimetazidine (TMZ) and diazoxide (DZ), significantly improved the survival rates and tissue disease in a lipopolysaccharide (LPS)-induced mouse model (69). Transplantation of bone marrow mesenchymal stem cells pretreated with lipopolysaccharide can regulate the immune response in septic mice and reduce inflammation (70). adipose mesenchymal stem cells (AD-MSCs) pretreated with eicosapentaenoic acid can reduce lung and distal organ damage, improve sepsis and increase the survival rates in cecal ligation and perforation(CLP)-induced experimental sepsis (71). Systemic administration of mesenchymal stem cells pretreated with interleukin-1β (β-mesenchymal stem cells) is more effective than untreated mesenchymal stem cells in improving the symptoms of sepsis in mice and increasing the survival rate. In addition, β-mesenchymal stem cells can effectively induce macrophages to differentiate into the anti-inflammatory M2 phenotype through paracrine activity (72). The purpose of staphylococcal enterotoxin B (SEB) pretreatment is to prolong the survival time of transplanted mesenchymal stem cells and induce the production of cytoprotective agents, antiapoptotic agents and anti-inflammatory factors by these cells. The downregulation of cytokine gene expression and the upregulation of the expression of antibacterial peptides and anti-inflammatory cytokines after transplantation indicate improvements in the therapeutic effects of SEB mesenchymal stem cells on live sepsis models (73).

### Regulatory Effects of Genetically Modified MSCs on Inflammation

At present, the treatment of various diseases with genetically modified mesenchymal stem cells mainly involves tissue repair, radiation injury therapy and tumor therapy. Some studies on the regulation of inflammation have shown that genetically modified MSCs not only have all the characteristics of stem cells but can also efficiently express foreign genes and enhance their anti-inflammatory effects in treating various diseases. BPI21/LL-37-modified human Umbilical Cord Mesenchymal Stem Cells (hUC-MSCs) exhibited significantly enhanced antibacterial and toxin-neutralizing activities *in vitro* (74). Bone marrow mesenchymal stem cells overexpressing TGF-β1 can reduce macrophage infiltration and induce macrophage transformation to enhance their therapeutic effects on organ damage and inflammation (75). In ulcerative colitis, the overexpression of IL-10 in mesenchymal stem cells enhanced their effects on *E. coli*-induced pneumonia and sepsis and enhanced the function of human macrophage (76). TLR3-activated mesenchymal plasma stem cells exhibit increased survival rates in response to sepsis induced by CLP, and the overexpression of miR-143 reduced the effectiveness of this treatment.

## The Safety and Efficacy of MSCs in Animal Experiments and Clinical Applications Associated With Sepsis and COVID-19

### The Effectiveness of MSCs in Animal Experiments of Sepsis

To date, no preclinical studies have shown that the application of bone marrow mesenchymal stem cells in animal models of sepsis has side effects. However, these results mainly come from rodent models and have limited correlations with human septicemia. Therefore, clinical applications are possible, it is necessary to thoroughly study the role of bone marrow mesenchymal stem cells in clinically relevant large animal models (77). Some studies have shown that a model of systemic inflammation induced by intravenous lipopolysaccharide can be used, and its safety has been verified. Although this model has limitations, it is necessary to further evaluate its safety and effectiveness for septic patients. However, early immunomodulation in an animal model of sepsis was confirmed. A preclinical study of a sepsis animal model showed that even if clinically related constructs were included, such as delayed administration of bone marrow mesenchymal stem cells after sepsis induction and combined administration of antibiotics and fluids, bone marrow mesenchymal stem cells could improve the survival rate (78). Bone marrow mesenchymal stem cells can reduce organ failure and help eliminate pathogens in the blood, peritoneum and spleen. The effectiveness of stem cell therapy has been examined in animals as models of human infection. Based on these findings, bone marrow mesenchymal stem cell treatment can inhibit the inflammatory reaction and the activation of nuclear factor kappa light chain enhancer, which activates B cells. Therefore, bone marrow mesenchymal stem cells may become a new treatment for acute lung injury caused by sepsis (15).

### The Safety and Efficacy of MSCs in Clinical Trials of Sepsis

The results of preclinical studies suggest that MSCs will become a new type of treatment for sepsis. Clinical studies have been performed, but there are currently only limited reports detailing the use of MSCs in humans, the number of cases is small, and there are still many ongoing clinical trials. A domestic researcher gave 15 patients with severe sepsis a single intravenous infusion of allogeneic bone marrow mesenchymal stem cells at doses of up to 3×10^6^ cells/kg. The treatment proved to be safe and well tolerated (70). A 72-year-old man was admitted with a diagnosis of COVID-19, ARDS, type-2 diabetes, diabetic nephropathy, renal insufficiency, and hypertension. Clinical treatment of severe pneumonia associated with COVID-19 with umbilical cord bone marrow mesenchymal stem cells has positive effects, delaying worsening of the disease and improving respiratory and renal functions (79). The results of the first phase of the Chinese Ischemic Stroke Subclassification (CISS) trial provide additional short-term data, showing that the administration of up to 250 million freshly cultured allogeneic bone marrow mesenchymal stem cells is safe for patients with septic shock. These data should give intensive care researchers additional reasons to continue the second phase of the trial to evaluate the efficacy and safety of bone marrow mesenchymal stem cells in the treatment of septic shock (80) (**Table 1**).

**Table 1:** Selected Clinical Studies Examining Effects MSCs of Relevance to Sepsis.

	NCT Number	Title	Status and Phase	Study Type and Results	Conditions	Outcome Measures:	Population
1	NCT02789995	Dysfunctions of Human Muscle Stem Cells in Sepsis	Completed Not Applicable	Interventional No Results Available	• Sepsis	• Muscle regenerative capacities • Satellite cell dysfunction after sepsis • Regenerative capacities of Human satellite cells in presence of mesenchymal stem cells	Enrollment:93 Age: 18 Years and older (Adult, Older Adult) Sex:All
2	NCT00100308	Unfractioned Heparin for Treatment of Sepsis	Completed Phase 3	Interventional No Results Available	• Sepsis • Bacterial Infections	• Change from baseline Multiple Organ Dysfunction (MOD) score • Length of stay • 28-day all-cause mortality	Enrollment:319 Age: 18 Years and older (Adult, Older Adult) Sex:All
3	NCT00357123	Effect of Rosuvastatin in Abdominal Sepsis	Unknown status Phase 2	Interventional No Results Available	• Sepsis	• Plasmatic levels of Interleukine 6 and 1B, and Tumor Necrosis Factor alpha (pg/dL) • Number of survivors • Plasmatic levels of Reactive C Protein (mg/dL) • Classification of severity by APACHE II scale • Incidence of complications or secondary effects	Enrollment:60 Age: 18 Years to 80 Years (Adult, Older Adult) Sex:All
4	NCT02370030	Effect of Citrulline on the Clinical and Biochemical Evolution of Patients With Sepsis.	Unknown status Phase 1 Phase 2	Interventional No Results Available	• Sepsis	Outcome Measures: Multiple organ failure	Enrollment:160 Age: 18 Years and older (Adult, Older Adult) Sex:All
5	NCT01315782	Alveolar Dead Space as Predictor of Organ Failure in Severe Sepsis	Recruiting Phase 2	Observational No Results Available	• Sepsis • Severe Sepsis • Septic Shock • Multi-organ Failure	• Multi-organ failure • Mortality	Enrollment:30 Age: 18 Years and older (Adult, Older Adult) Sex:All
6	NCT03882476	RCT of Sepsis Machine Learning Algorithm	Not yet recruiting	No Results Available	• Sepsis • Severe Sepsis • Septic Shock	In-hospital SIRS-based mortality	Enrollment:51645 Age: 18 Years and older (Adult, Older Adult) Sex:All
7	NCT03644940	Subpopulation-Specific Sepsis Identification Using Machine Learning	Not yet recruiting Phase 2	Interventional No Results Available	• Sepsis • Severe Sepsis • Septic Shock	• In-hospital SIRS-based mortality • In-hospital severe sepsis/shock- coded mortality • SIRS-based hospital length of stay • Severe sepsis/shock-coded hospital length of stay	Enrollment:51645 Age: 18 Years and older (Adult, Older Adult) Sex:All
8	NCT03734484	Gram Type Infection-Specific Sepsis Identification Using Machine Learning	Not yet recruiting Phase 2	Interventional No Results Available	• Sepsis • Severe Sepsis • Septic Shock	• Change in time to antibiotic administration • Change in administration of unnecessary antibiotics	Enrollment:51645 Age: 18 Years and older (Adult, Older Adult) Sex:All
9	NCT04005001	HindSight Phase II	Not yet recruiting Phase 2	Interventional No Results Available	• Sepsis • Severe Sepsis • Septic Shock	False alert reduction	Enrollment:51645 Age: 18 Years and older (Adult, Older Adult) Sex:All
10	NCT03369275	Cellular Immunotherapy for Septic Shock	Unknown status Phase 2	Interventional No Results Available	• Septic Shock • Sepsis • Pathologic Processes • Shock • Infection • Systemic Inflammatory Response Syndrome • Inflammation	• The reduction in days on mechanical ventilation, or renal replacement therapy, or vasopressors. • Incidence of treatment-emergent adverse events (Safety and tolerability) • Biological endpoints as markers of vascular permeability • Mortality • Organ Failure Scores • Organ Support Measures • Length of ICU Stay (in days) • Length of Hospital Stay (in days)	Enrollment:114 Age: 18 Years and older (Adult, Older Adult) Sex:All

### Examples of MSCs Applications in COVID-19 Patients

A total of 88 trials were registered to study the safety and efficacy of stem cells in treating COVID-19. The indications under investigation include COVID-19 pneumonia, severe pneumonia, respiratory failure, acute respiratory distress syndrome and pulmonary fibrosis. Most of the studies are aimed at treating patients with “COVID-19 pneumonia” (19 out of 88 cases) and “severe/severe pneumonia” (37 out of 88 cases). According to a meta-analysis of 50,466 inpatients with pneumonia associated with COVID-19, 14.8% of pneumonia patients with COVID-19 developed acute respiratory distress syndrome (73). Among 88 studies, 24 studies investigated the treatment of patients with acute respiratory distress syndrome. Although patients with acute respiratory distress syndrome often show pulmonary fibrosis after discharge (75), out of 88 studies, only 2 were registered to study the curative effect of stem cell therapy on patients with pulmonary fibrosis. Interestingly, only one of the 88 studies used “*in vitro* stromal cell therapy” to treat COVID-19 pneumonia patients with acute kidney injury. Most of these clinical trials (63 out of 88) tested the feasibility, tolerance and safety of serious adverse events associated with stem cell therapy (19 trials in Phase I, 24 trials in Phase I/II and 20 trials in Phase II). Few clinical trials have progressed beyond the second stage (3.4%), only two trials are in the second/third stage, and only one trial is in the third stage. In 22 studies, the clinical stage was unclear or “not applicable”.

The latest research shows that (81) Umbilical Cord Mesenchymal Stem Cells (UC-MSCs) infusions in COVID-19 ARDS patients were safe. Inflammatory cytokines were significantly decreased in UC-MSC-treated subjects at day 6 (**Table 2**).

**Table 2:** Selected Clinical Studies Examining Effects MSCs of Relevance to COVID-19.

	NCT Number	Title	Status	Study Results	Conditions	Interventions	Study Type and Phase	Characteristics	Population	Dates
1	NCT04313322	Treatment of COVID-19 Patients Using Wharton's Jelly-Mesenchymal Stem Cells	Recruiting	No Results Available	• Use of Stem Cells for COVID-19 Treatment	• Biological: WJ-MSCs	Interventional Phase 1	• Clinical outcome • CT Scan • RT-PCR results	Enrollment:5 Age:18 Years and older (Adult, Older Adult) Sex:All	Primary Completion: June 30, 2020
2	NCT04625738	Efficacy of Infusions of MSC From Wharton Jelly in the SARS-Cov-2 (COVID-19) Related Acute Respiratory Distress Syndrome	Not yet recruiting	No Results Available	• COVID19 ARDS	• Biological: Ex vivo expanded Wharton's Jelly Mesenchymal Stem Cells • Biological: Placebo	Interventional Phase 2	• PaO2 / FiO2 ratio • respiratory function evolution • respiratory assistance • organ failures 1 • organ failures 2 • organ failures 3 • duration of intensive care • Cause of death • respiratory morbidity (TDM, functional respiratory measures) • viral load • and 5 more	Enrollment:30 Age:18 Years and older (Adult, Older Adult) Sex:All	Primary Completion: May 16, 2022
3	NCT04252118	Mesenchymal Stem Cell Treatment for Pneumonia Patients Infected With COVID-19	Recruiting	No Results Available	• COVID-19	• Biological: MSCs	Interventional Phase 1	• Size of lesion area by chest radiograph or CT • Side effects in the MSCs treatment group • Improvement of Clinical symptoms including duration of fever and respiratory • Time of nucleic acid turning negative • Rate of mortality within 28-days • CD4+ and CD8+ T celll count • Alanine aminotransferase • C-reactive protein • Creatine kinase	Enrollment:20 Age:18 Years to 70 Years (Adult, Older Adult) Sex:All	Primary Completion: December 2020
4	NCT04444271	Mesenchymal Stem Cell Infusion for COVID-19 Infection	Recruiting	No Results Available	• COVID-19	• Drug: Mesenchymal stem cells • Other: Placebo	Interventional Phase 2	• Overall survival • Clinical improvement • Time of COVID19 PCR negativity • Radiological improvement (day 15 and day 30 assessment) • days required to discharge from hospital	Enrollment:20 Age:10 Years and older (Child, Adult, Older Adult) Sex:All	Primary Completion: August 30, 2020
5	NCT04339660	Clinical Research of Human Mesenchymal Stem Cells in the Treatment of COVID-19 Pneumonia	Recruiting	No Results Available	• COVID-19	• Biological: UC-MSCs • Other: Placebo	Interventional • Phase 1 • Phase 2	• The immune function • Blood oxygen saturation • Rate of mortality within 28-days • Size of lesion area by chest imaging • CD4+ and CD8+ T cells count • Peripheral blood count recovery time • Duration of respiratory symptoms (fever, dry cough, difficulty breathing, etc.) • COVID-19 nucleic acid negative time	Enrollment30 Age:18 Years to 75 Years (Adult, Older Adult) Sex:All	Primary Completion: June 30, 2020
6	NCT04399889	hCT-MSCs for COVID19 ARDS	Recruiting	No Results Available	• COVID • Corona Virus Infection • COVID19	• Biological: human cord tissue mesenchymal stromal cells	Interventional • Phase 1 • Phase 2	Outcome Measures: • Safety of the Investigational Product • Describe the potential for MSC therapy to favorably alter the course of COVID-ARDs	Enrollment:30 Age:18 Years and older (Adult, Older Adult) Sex:All	Primary Completion: April 1, 2021
7	NCT04273646	Study of Human Umbilical Cord Mesenchymal Stem Cells in the Treatment of Severe COVID-19	Not yet recruiting	No Results Available	• 2019 Novel Coronavirus Pneumonia • COVID-19	• Biological: UC-MSCs • Drug: Placebo	Study Type: Interventional Phase: Not Applicable	• Pneumonia severity index • Oxygenation index (PaO2/FiO2) • Side effects in the UC-MSCs treatment group • 28-days survival • Sequential organ failure assessment • C-reactive protein • Procalcitonin • Lymphocyte count • CD3+, CD4+ and CD8+ T celll count • CD4+/CD8+ratio	Enrollment:48 Age:18 Years to 65 Years (Adult, Older Adult) Sex:All	Primary Completion: June 30, 2020
8	NCT04397796	Study of the Safety of Therapeutic Tx With Immunomodulatory MSC in Adults With COVID-19 Infection Requiring Mechanical Ventilation	Recruiting	No Results Available	• COVID	• Biological: BM-Allo.MSC • Biological: Placebo	Study Type: Interventional Phase: Phase 1	• Incidence of AEs • Mortality • Death • Number of ventilator-free days • Improvement of one category • 7-point ordinal scale • NEWS • NEWS of # 2 • Sequential Organ Failure Assessment (SOFA) • Oxygen • Hospitalization • Incidence of SAEs	Enrollment:45 Age:18 Years to 80 Years (Adult, Older Adult) Sex:All	Primary Completion: June 2021
9	NCT04346368	Bone Marrow-Derived Mesenchymal Stem Cell Treatment for Severe Patients With Coronavirus Disease 2019 (COVID-19)	Not yet recruiting	No Results Available	• Coronavirus Disease 2019 (COVID-19)	• Biological: BM-MSCs • Biological: Placebo	Study Type: Interventional Phase: • Phase 1 • Phase 2	• Changes of oxygenation index (PaO2/FiO2) • Side effects in the BM-MSCs treatment group • Clinical outcome • Hospital stay • CT Scan • Changes in viral load • Changes of CD4+, CD8+ cells count and concentration of cytokines • Rate of mortality within 28-days • Changes of C-reactive protein	Enrollment: 20 Age:18 Years to 75 Years (Adult, Older Adult) Sex: All	Primary Completion: December 2020
10	NCT04537351	The Mesenchymal COVID-19 Trial: a Pilot Study to Investigate Early Efficacy of MSCs in Adults With COVID-19	Recruiting	No Results Available	• Covid19 • Acute Respiratory Distress Syndrome	• Biological: CYP-001	Interventional • Phase 1 • Phase 2	• Trend in trajectory of PaO2/FiO2 ratio (P/F ratio) between groups • Incidence and severity of treatment- emergent adverse events • Change in C-reactive protein (CRP) levels • Proportional differences between groups on the Clinical Improvement Scale • Changes in P/F ratio • Changes in respiratory rate • Changes in oxygenation index • Changes in respiratory compliance (the change in lung volume per unit change in transmural pressure gradient) • Changes in positive end-expiratory pressure • Ventilator-free days • Proportional differences between groups on the SF-36 • Proportional differences between groups on the mini mental state examination	Enrollment:24 Age:18 Years and older (Adult, Older Adult) Sex: All	Primary Completion: March 31, 2021

## Summary and Outlook

In the face of sepsis and COVID-19 pneumonia patients and their serious complications, such as septic shock, acute respiratory distress syndrome and multiple organ dysfunction syndrome, traditional therapy is powerless. Preclinical and preliminary clinical data show that MSCs can reduce lung injury caused by an excessive immune response to viruses and reduce the risk of CSS through anti-inflammatory and immunomodulatory effects.

Despite the progress in the field, drug discovery of inflammatory diseases remains a major challenge due to the limited understanding of specific inflammatory responses in different pathologies (82). The recent emergence of new viruses such as SARS-COV-2 (83) have triggered a number of popular pandemics, which poses an unprecedented threat to global public health. Although significant progress has been made in the direction of nucleic acid detection, neutralizing antibody and vaccine development, it is still variable to reduce effective strategies of cytokine storm in COVID-19 (84) severe patients. Mainly due to the complexity of cytokines and the diversity of targets, the intervention of a single cytokine is not sufficient to relieve inflammatory responses. The non-specific biodistribution *in vivo* and dose-limited side effects further limit the wide application of these free antibodies. The latest developments in biomaterials and nanotechnology (85) provide many promising opportunities for infectious and inflammatory diseases.Recently, the team of Rao (86) and other researchers proposed new biomaterials in improving the role of antibody and broad spectrum cytokines. This multi-functional neutralization platform (87) can solve the problem of multiple cytokines released in different diseases at the same time, which is conducive to the development. A new therapy to deal with cytokine storms caused by sepsis (88) and COVID-19 (89) and other inflammatory diseases.

In addition, the current research shows that treatment with bone marrow mesenchymal stem cells is safe and effective. However, the existing research has limitations, such as a small sample size and short follow-up time, which are not convincing and limit its clinical application. Therefore, it is necessary to further verify the effectiveness, safety and corresponding mechanism of bone marrow mesenchymal stem cells in the treatment of COVID-19 pneumonia through large-scale, multifocal, multicenter and long-term follow-up studies. In addition, the timing, dosage, indications, contraindications and adverse reactions of mesenchymal stem cell infusion should be further examined.

## Author Contributions

HK was responsible for hypothesis generation. HK and ZQ were responsible for the conception of this study. LW, YZ, and MY contributed to study design and data interpretation. LW, YL, and MX were responsible for writing the article. YYL, RY, and HW performed data acquisition. YZ, YS, and HZ conducted data analysis. All authors contributed to the article and approved the submitted version.

## Conflict of Interest

The authors declare that the research was conducted in the absence of any commercial or financial relationships that could be construed as a potential conflict of interest.

## Publisher’s Note

All claims expressed in this article are solely those of the authors and do not necessarily represent those of their affiliated organizations, or those of the publisher, the editors and the reviewers. Any product that may be evaluated in this article, or claim that may be made by its manufacturer, is not guaranteed or endorsed by the publisher.

## Abbreviations

MSCs, Mesenchymal stem cells; BMSCs, Bone marrow mesenchymal stem cells; WJ-MSCs, Wharton’s jelly mesenchymal stem cells; hESC-MSCs, Human embryonic stem cell derived mesenchymal stem cells; hUC-MSCs, Human umbilical cord mesenchymal stem cells; UC-MSCs,Umbilical cord mesenchymal stem cells; AD-MSCs, Adipose derived mesenchymal stem cells; COVID-19, Coronavirus disease 2019; CSS, Cytokine storm syndrome; ALI, Acute lung injury; SARS-CoV-2, Severe acute respiratory syndrome coronavirus 2; ARDS, Acute respiratory distress syndrome; MERS, Middle East respiratory syndrome; TNF-α, Tumor necrosis factor-α; sTNFR1, Soluble tumor necrosis factor receptor; TGF-β, Transforming growth factor-β; TLR3, Toll-like receptor; HGF, Hepatocyte growth factor; IL, Interleukin; AMs, Alveolar macrophages; MIP-1α, Macrophage inflammatory protein-1α; IFN-α, Interferon-α; IFN-γ, Interferon-γ; IDO, Indoleamine 2,3-dioxygenase; NO, Nitric oxide; iNOS, Inducible nitric oxide synthase; PGE2, Prostaglandin E2; CXCR, Chemokine receptor; CXCL12, Chemokine receptor; IgM, Immunoglobulin M; IgG, Immunoglobulin G; IgA, Immunoglobulin A; EVs, Extracellular vesicles; NK, Natural killer; NKp30, Natural-killer group 2, member D; NKp30, Natural killer protein 30; NKp44, Natural killer protein 44; MIF, Migration inhibitory factor; PRRs, Pattern recognition receptors; ssRNA, Single-Stranded Ribonucleic Acid; CTLs, Cytotoxic T lymphocytes; WBC, White blood cell; Th, Helper T lymphocytes; ROS, Reactive oxygen species; CAP, Choline anti-inflammatory pathway; a7nAChR, A7 nicotinic acetylcholine receptor; HO-1, Heme oxygenase 1; TMZ, Trimetazidine; DZ, Diazoxide; LPS, Lipopolysaccharide; CLP, Cecal ligation and perforation; SEB, Staphylococcal enterotoxin B; Bcl-2, B-cell lymphoma-2; ROS, reactive oxygen species; CISS, Chinese Ischemic Stroke Subclassification.
